# Double-Hit Diffuse Large B-Cell Lymphoma in AIDS

**DOI:** 10.7759/cureus.73190

**Published:** 2024-11-07

**Authors:** Subha Sree S, Swathy Moorthy

**Affiliations:** 1 General Medicine, Sri Ramachandra Medical College, Sri Ramachandra Institute of Higher Education and Research, Chennai, IND; 2 Internal Medicine, Sri Ramachandra Medical College, Sri Ramachandra Institute of Higher Education and Research, Chennai, IND

**Keywords:** aids, gastric lymphoma, hiv infection, large b cell lymphoma, tuberculosis in hiv

## Abstract

Diffuse large B-cell lymphoma (DLBCL) is the most common type of immunoblastic lymphoma associated with AIDS, with the stomach being the most frequent extranodal site of involvement. Despite the widespread use of combined antiretroviral therapy (cART), the incidence of systemic lymphomas remains relatively high. These lymphomas often present in the early stages of AIDS as high-grade malignancies. We report the case of a man in his early 30s who initially presented with chronic cough and weight loss, diagnosed with pulmonary tuberculosis and found to be HIV-positive. Within a few months, he returned with persistent abdominal pain and progressive weight loss. Imaging revealed a gastric ulcer, and biopsy confirmed the diagnosis of DLBCL. Although cART is now available and started early upon an HIV diagnosis, vigilant surveillance, early diagnosis, and prompt initiation of chemotherapy are critical for achieving an adequate response and remission.

## Introduction

Impaired immunosurveillance due to diminished CD4+ counts, a hallmark of AIDS, contributes to the development of AIDS-defining malignancies such as Kaposi sarcoma, non-Hodgkin’s lymphoma (NHL), and invasive cervical carcinoma [1], while also increasing the incidence of non-AIDS-defining malignancies like Hodgkin’s disease, leukemia, multiple myeloma, and other solid organ tumors [2]. The lifetime risk of NHL in the general population is approximately 1 in 108 for men and 1 in 162 for women [3], although this risk is significantly higher in people living with HIV/AIDS (PLHA) [2]. While the advent of combined antiretroviral therapy (cART) has significantly reduced the morbidity and mortality associated with AIDS-defining malignancies [4,5], the incidence of systemic lymphomas remains comparatively high, with immunoblastic lymphomas accounting for 60% of cases. Aggressive diffuse large B-cell lymphoma (DLBCL) is the most common type of immunoblastic lymphoma in PLHA and is typically high-grade. These lymphomas often present in the early stages of AIDS, predominantly involving the gastrointestinal tract in extranodal sites. Despite being the most common systemic lymphoma, its incidence has decreased significantly, from 63% in the pre-cART era to 35-37% in the post-cART era [6]. The availability of more effective cART regimens with minimal drug interactions has shown promising results in the treatment of systemic lymphomas, particularly when used in conjunction with standard chemotherapy regimens such as cyclophosphamide, doxorubicin, vincristine, and prednisolone (CHOP) and dose-adjusted etoposide, prednisone, vincristine, cyclophosphamide, and hydroxydaunorubicin, resulting in improved survival rates [7-9]. The introduction of rituximab, an anti-CD20 monoclonal antibody, to the standard CHOP regimen has significantly improved survival rates in the general population, although its use in HIV remains controversial due to an increased risk of infection-related deaths [10,11].

## Case presentation

**Figure 1:**
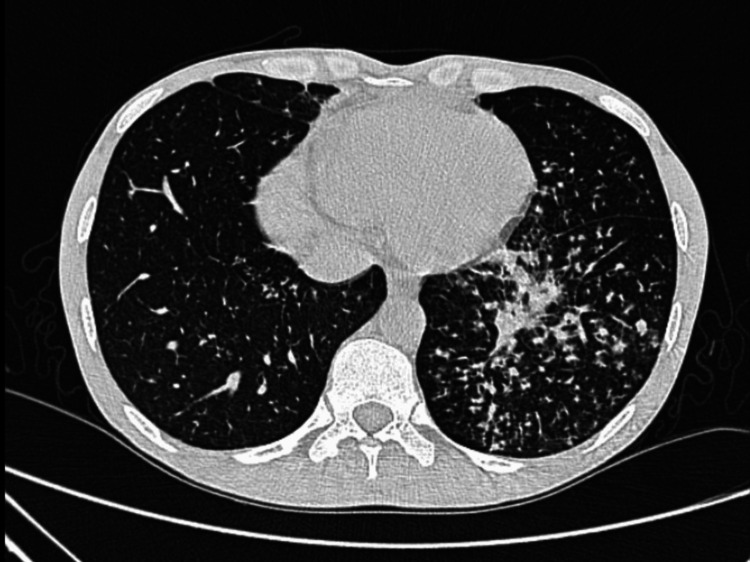
HRCT image showing ill-defined centrilobular nodules with a tree-in-bud appearance, some coalescing to form a consolidative patch, suggestive of active pulmonary tuberculosis HRCT, high-resolution CT

A man in his early 30s, a chronic smoker for the past 12 years with 10 pack years, presented with complaints of low-grade fever, cough with scanty mucoid expectoration, loss of appetite, significant unintentional weight loss of about 10-15 kg, and diminished hearing over a period of two months. On examination, the patient was emaciated and had multiple subcentimetric to 1.5 cm-sized palpable, nontender cervical, axillary, and inguinal lymph nodes. Coarse crepitations were heard over the bilateral supra- and infraclavicular regions, and the rest of his examination was unremarkable. High-resolution CT thorax showed features suggestive of active pulmonary tuberculosis (Figure 1) and mediastinal lymphadenopathy. His sputum smear tested positive for acid-fast bacilli, and Genexpert detected Mycobacterium tuberculosis (MTB), rifampicin-sensitive. The patient was started on a weight-based antitubercular treatment regimen. HIV screening was reactive, and the absolute CD4 count was found to be 125. He was started on a prophylactic dose of cotrimoxazole double strength (160/800 mg) once daily, and workup for other opportunistic infections and AIDS-defining illnesses was negative. Dolutegravir-based antiretroviral therapy was initiated two months after starting antitubercular treatment. The patient showed clinical improvement and was discharged.

**Figure 2:**
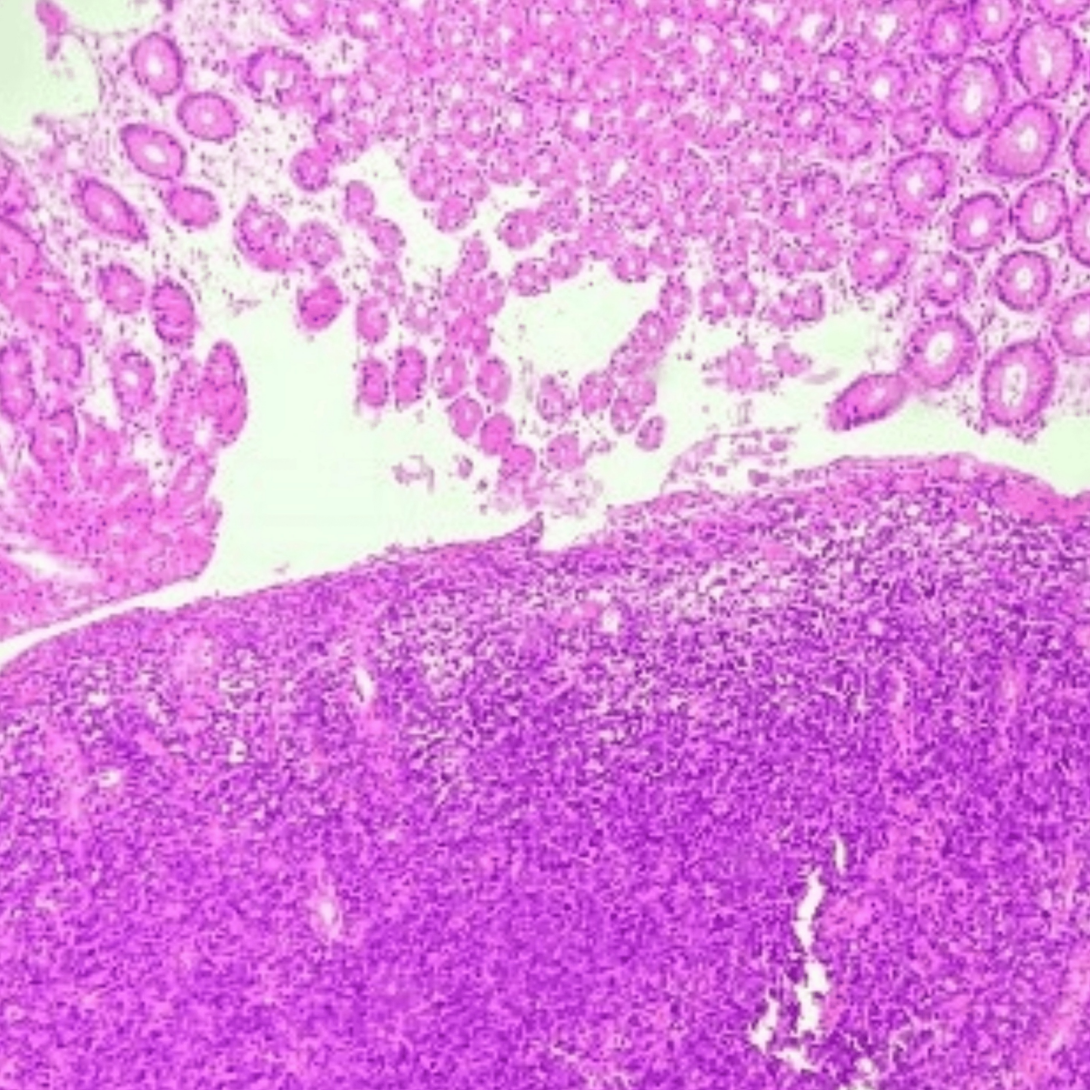
Gastric ulcer biopsy showing sheets of atypical cells

**Figure 3:**
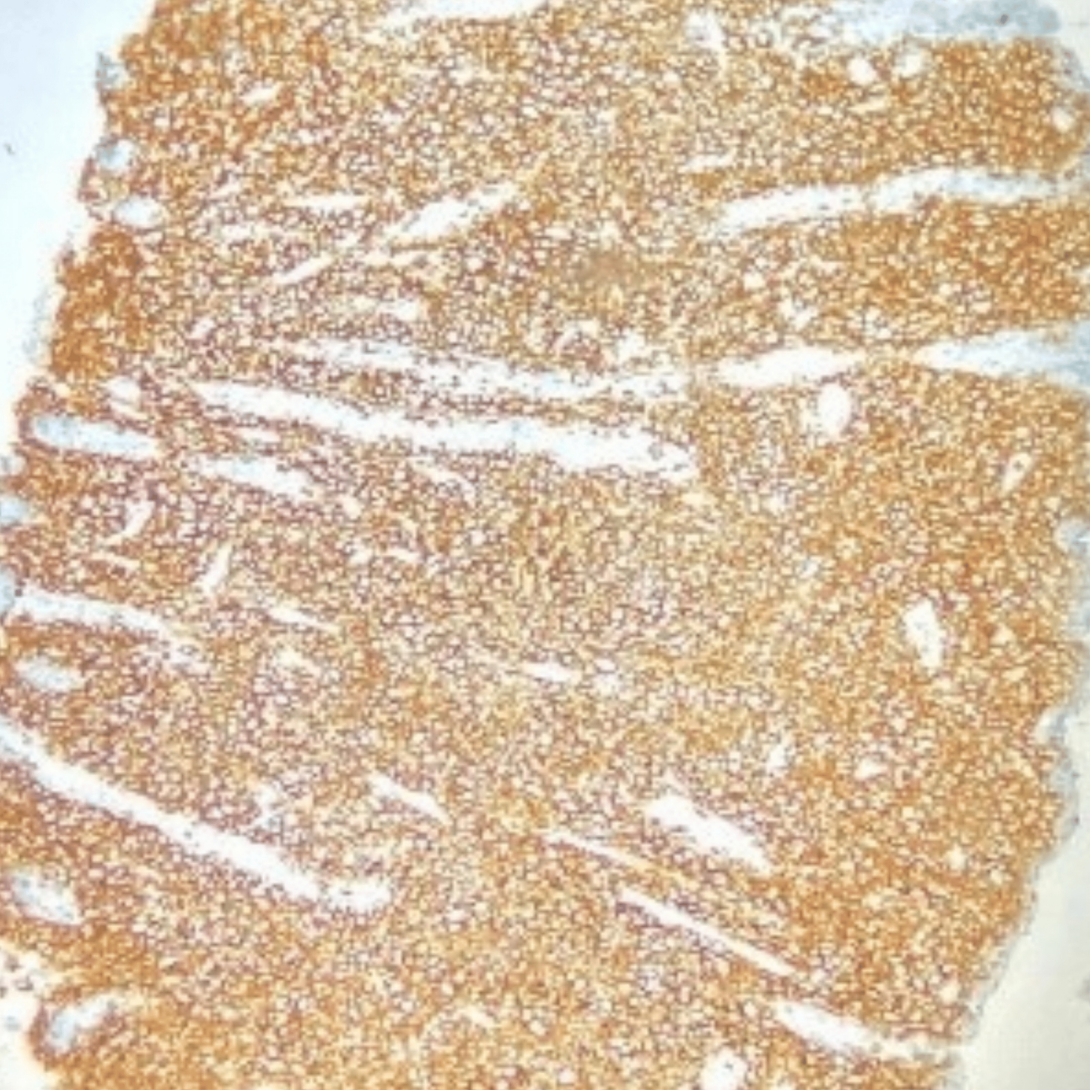
IHC showing strong positivity for CD45 IHC, immunohistochemistry

**Figure 4:**
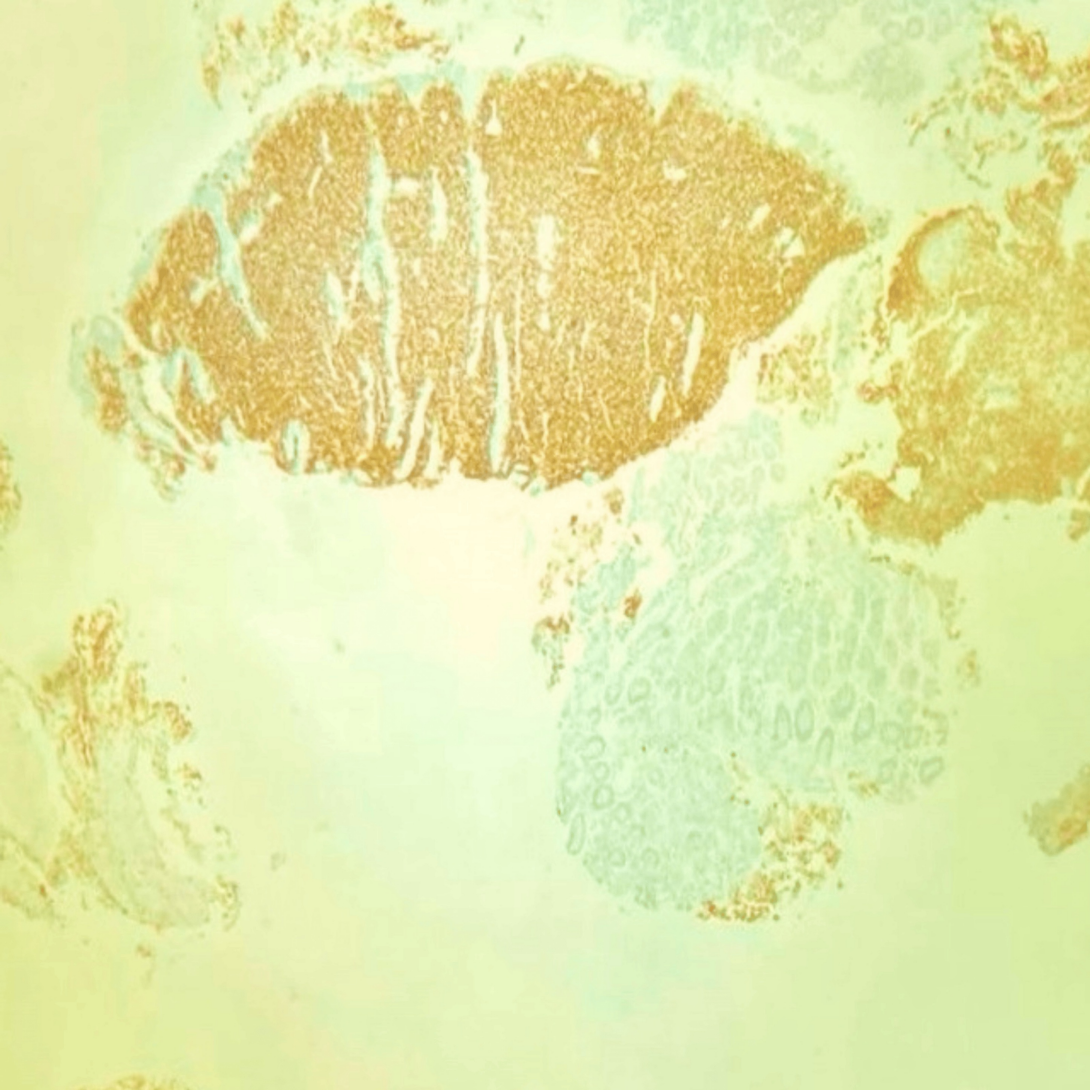
IHC showing strong positivity for CD20 IHC, immunohistochemistry

**Figure 5:**
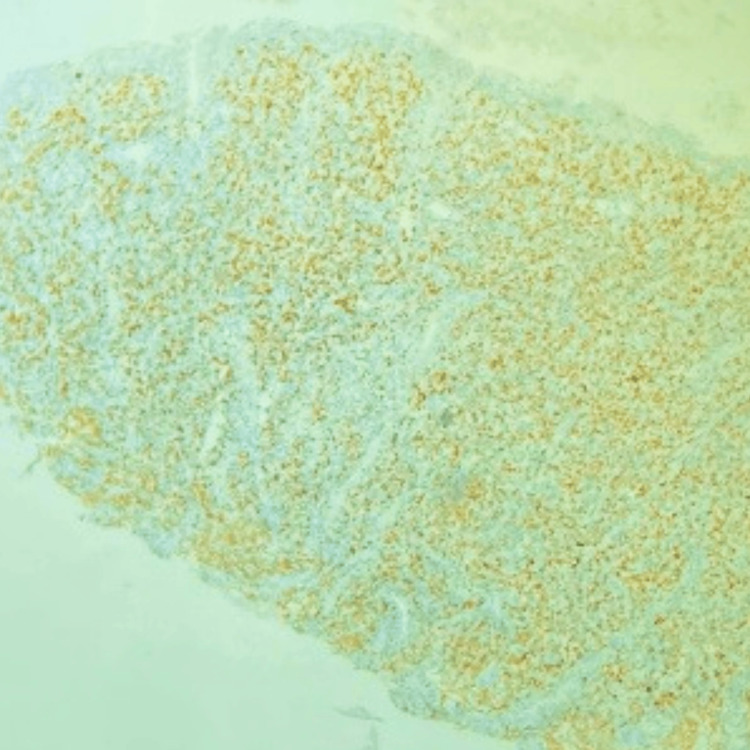
IHC showing strong positivity for c-MYC IHC, immunohistochemistry

**Figure 6:**
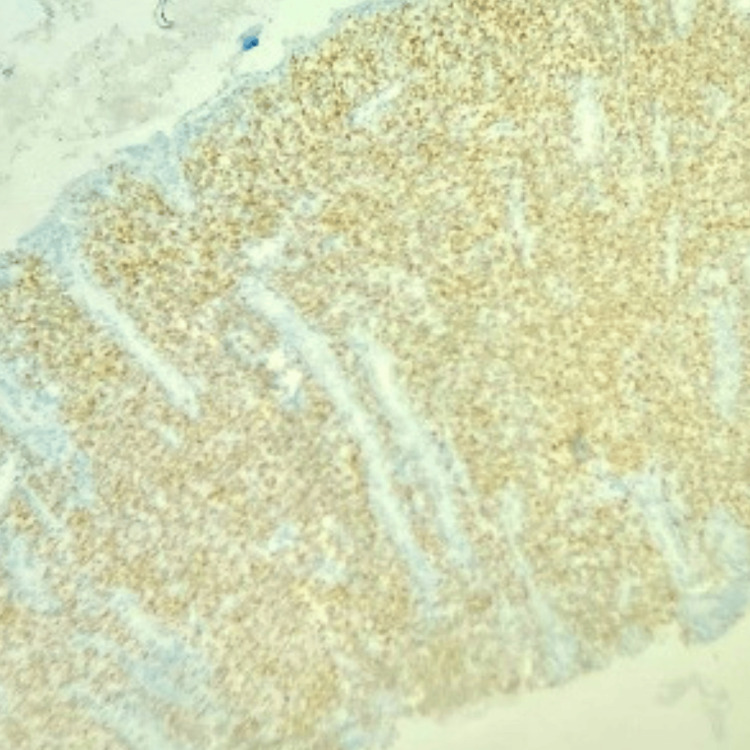
IHC showing strong positivity for MUM1 IHC, immunohistochemistry

**Figure 7:**
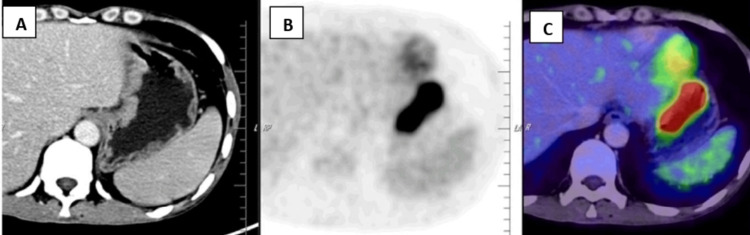
FDG PET scan showing avid, ill-defined wall thickening along the lesser curvature of the cardia, body, and adjacent antrum of the stomach, with suspicious periserosal infiltration and loss of fat plane with the left lobe of the liver (A) Irregular wall thickening along the lesser curvature of the stomach. (B) Increased radiotracer uptake. (C) Metabolically active lesion along the lesser curvature of the stomach, SUV max 23. FDG, fluorodeoxyglucose

During a follow-up review seven months after the initiation of treatment, he presented with complaints of diffuse abdominal pain associated with melena, three to four episodes per week. Upper gastrointestinal endoscopy revealed grade I monilial esophagitis, a severely inflamed stomach, and a large gastric ulcer in the fundus of the stomach with multiple bulbar ulcers and post-bulbar narrowing. A biopsy was taken, and the patient was started on proton pump inhibitors infusion. The biopsy revealed poorly differentiated DLBCL with tumor cells strongly positive for CD45, CD20, BCL6, MUM-1, and c-Myc (80%) (Figure 2, Figure 3, Figure 4, Figure 5, Figure 6), and negative for BCL2, cyclin D1, and CD10, representing a double-expressor type of non-germinal center DLBCL. A whole-body PET-CT done for the staging of lymphoma revealed multifocal malignant-looking wall thickening along the lesser and greater curvature of the stomach (Figure 7), two jejunal loops, bilateral fluorodeoxyglucose (FDG) avid lymphadenopathy, and non-FDG avid lymphadenopathy along the lesser and greater curvatures of the stomach and in the para-aortic region. Bone marrow aspiration and biopsy revealed hypercellular marrow. The patient was then initiated on the rituximab-CHOP (R-CHOP) regimen and continued with dolutegravir-based ART. The patient completed six cycles of the prescribed chemotherapy and continues to do well, taking dolutegravir-based ART. He has remained on follow-up for the last four years.

## Discussion

MTB may be associated with the subsequent development of malignancies, as it creates an appropriate microenvironment for cancer progression through chronic inflammation [12]. NHL has been found to be preceded by chronic inflammatory conditions, as evidenced by the development of NHL following hepatitis C, *Campylobacter jejuni*, and *Helicobacter pylori* infections [13]. Tuberculosis and HIV coinfections potentiate one another, accelerating the deterioration of immunocompetence, leading to the early onset of malignancies and an increased risk of opportunistic infections, which ultimately escalates morbidity and mortality, as seen in our patient. Loss of immunosurveillance and persistent antigenic stimulation from HIV antigens, including gp120, p17, and TAT, provoke uncontrolled chronic B cell activation, which in turn promotes lymphomagenesis [14]. Among the two morphological variants, the immunoblastic variant is more commonly associated with HIV-related DLBCL (Table 1) [15]. These cases are strongly positive for the plasma cell surface marker CD138, while mature B cell markers, such as CD45 and CD20, are typically down-regulated [16].

**Table 1:** DLBCL morphological variants in PLHA: pathologic and immunophenotype markers, virologic coinfection, and genetic features Adapted from Huguet et al. (2023) [5]

DLBCL subtype	CD20	CD138	BCL6	MUM1	Other mutations
Immunoblastic	+/-	+	-	+	CD10, CD5, CD30
Centeroblastic	+	-	+	-	CD10, CD5

In our patient, there is strong positivity for CD45 and CD20. The expression of MUM1 in the biopsy corresponds to the activated B cell/nongerminal center subtype. Additionally, the tumor is strongly positive for both MYC and BCL6, classifying it as double-hit lymphoma, or the double-expressor type, which is associated with an extremely poor prognosis and a median survival of 12-18 months [17]. DLBCL in the context of AIDS is typically of higher grade, with up to 40% being extranodal, and it often presents at an advanced clinical stage with poor prognosis [18]. Among extranodal sites, the gastrointestinal tract is the most common, and in this case, the presenting symptom was an aggressive double-expressor subtype of DLBCL manifested as a gastric ulcer.

Prior to initiating chemotherapy, a staging procedure, such as a baseline 18-Fluoro-2-deoxy-D-glucose PET scan, is recommended to improve staging accuracy. In our patient, the scan revealed stage II E lymphoma. A trephine biopsy is also needed to rule out bone marrow involvement. The prevalence of Epstein-Barr virus (EBV) infection is reported to be 90-100% in the immunoblastic variant of DLBCL in HIV, compared to 25-30% in the centroblastic variant [2,16]; however, our patient was not evaluated for EBV due to financial constraints.

The patient was initiated on the highly effective R-CHOP regimen, as studies have shown improved complete response rates [7-9], two-year progression-free survival rates [7,9], and two-year overall survival rates [19], with a reduced rate of infectious death. A dolutegravir-based regimen, an unboosted integrase strand transfer inhibitor (INSTI), was used as concomitant therapy, which has demonstrated better complete response rates and improved immune status post-chemotherapy [20]. While drug-drug interactions are a concern when chemotherapy and cART are used together, our patient responded well to the chemotherapy regimen and completed 6 cycles, achieving a disease progression-free recovery period.

## Conclusions

In patients with aggressive DLBCL, HIV should be suspected, and appropriate screening and evaluation should be conducted to enable the administration of effective chemotherapy alongside a highly active cART regimen, thereby improving prognosis and remission rates. INSTI-based regimens should be preferred to minimize potential drug-drug interactions, enhancing the effectiveness of both concomitant chemotherapy and antiretroviral therapy. PLHA, particularly those with low CD4 counts and reduced immune surveillance, should be regularly screened for aggressive AIDS-defining malignancies to facilitate early diagnosis and efficient management.
